# The perspectives of parents/carers on a new parental education occupational therapy intervention

**DOI:** 10.1177/03080226251404423

**Published:** 2025-12-26

**Authors:** Teresa Joyce, Anna L. Barnett

**Affiliations:** 1 School of Health and Care, Coventry University, London, UK; 2 School of Psychology, Social Work and Public Health, Oxford Brookes University, Oxford, UK

**Keywords:** intervention, parental education, ADHD, DCD, ASD, neurodevelopmental disorders

## Abstract

**Introduction:**

Parental education has been highlighted as an effective intervention for children, young people and families. However, no parental education interventions are underpinned by occupational therapy (OT) theory. This study aimed to understand the perspectives of parents/carers of children with neurodevelopmental disorders on the acceptability and design of a parental educational intervention underpinned by OT theory.

**Method:**

23 parents/carers of children aged between 5 and 11 years who had a diagnosis of Autism Spectrum Disorder, Attention Deficit Hyperactivity Disorder and/or Developmental Coordination Disorder participated in one of three online 90-minute focus groups. Participants were presented with a draft of the proposed intervention and engaged in a facilitated discussion about the acceptability and feasibility of the intervention. Data were analysed using thematic analysis.

**Results:**

Four themes were generated: (1) Parents want to develop skills to support their child, (2) parents also need support, (3) there are barriers to reaching parents and (4) parents need an inclusive environment to promote their learning.

**Conclusion:**

Parents/carers acknowledged the benefits of the intervention, highlighting the importance of focusing on supporting parents as well as their children. They also raised potential barriers to participation, which included time and money, and emphasised the importance of designing the group with inclusivity as a central principle.

## Background

Occupational therapists work with children, young people and their families to support participation in everyday life. Occupational therapy (OT) intervention can be divided into three tiers: Specialist, in which an occupational therapist delivers 1:1 intervention with a child or family, Targeted, in which an occupational therapist supports a small group to achieve occupational participation, and Universal, in which occupational participation is achieved on a wider scale (e.g. a whole school) by incorporating principles such as universal design (Royal College of Occupational Therapists (RCOT), 2022).

Traditional specialist models of OT intervention delivery, in which an ‘expert therapist’ delivers 1:1 intervention with a child, can promote dependency and disempower families (RCOT, 2022). Coaching and parental education interventions provide an opportunity to empower families to independently solve challenges in everyday life, reducing dependence on services. In a systematic review, Novak and Honan (2019) categorised both approaches as ‘effective interventions’. Coaching is a specialist-level intervention where occupational therapists partner with families to achieve occupational goals (Kessler and Graham, 2015). The occupational therapist’s role is to support families through asking questions and prompting reflection. Some approaches include teaching or modelling (Kessler and Graham, 2015); however, teaching is not the focus. By contrast, parental education via a therapist-led group is a targeted level intervention. Working in groups is more cost-effective and provides opportunities for friendships and emotional support (Cole, 2018). However, current parental education programmes often focus exclusively on behaviour management (Barlow et al., 2012) or reducing parental stress through mindfulness (Dykens et al., 2014). Alternatively, they focus on just one area, such as the communication skills of the child (Tanner et al., 2015).

As Novak and Honan (2019) have highlighted that parental education is an effective intervention for children and young people, an occupational therapist should look to utilise this method to support children and young people. However, there are currently no documented parental education programmes underpinned by OT theory (Novak and Honan, 2019). A proposed novel intervention would be a targeted level parental education intervention for parents of children with neurodevelopmental disorders. Neurodevelopmental disorders are a group of conditions present from the early developmental period and are associated with impaired cognition, communication, adaptive behaviour and/or psychomotor skills (American Psychiatric Association, 2013). Autism Spectrum Disorder (ASD), Attention Deficit Hyperactivity Disorder (ADHD) and Developmental Coordination Disorder (DCD) are all Neurodevelopmental Disorders that commonly co-occur (Lino and Chieffo, 2022).

This proposed parental education intervention would focus on teaching parents/carers reasoning informed by the Person-Environment-Occupation-Performance (PEOP) model (Baum et al., 2015). Parents/carers will gain skills to independently assess barriers to occupational performance and make environmental and task adaptations to increase participation across a range of occupations. For example, consider a child who is having difficulties riding a bicycle in a busy park with uneven surfaces. Parents will learn about assessing and intervening at the level of the person, the environment and the occupation. Intervening at the level of the environment could include going to a quieter park and using one even stretch of tarmac. An occupational intervention could be using an adapted bike or trike, making the task more achievable at the child’s current skill level. Intervening at the level of the person could include task-specific instruction to develop the child’s ability to ride a bicycle independently (a further worked example of this model in practice is provided in Supplemental 2).

The first stage of developing a new intervention is to engage with stakeholders to understand if the proposed intervention is something that is needed and desired and to consider key content and design principles (Skivington et al., 2021). Therefore, this study aimed to gain the perspectives of parents/carers of children with neurodevelopmental disorders on the acceptability and design of a parental educational intervention underpinned by OT theory. This project aligns with two Royal College of Occupational Therapists (RCOT, 2019) research priorities by (1) considering the role of OT in supporting self-management and (2) using co-production to consider how to effectively work with families.

## Method

Online focus groups were chosen to collect qualitative data to gain parents/carers’ views on the initial conception of a new OT parental education intervention. Including parents/carers from this early stage of intervention development is aligned with recommendations regarding the development of complex interventions (Skivington et al., 2021). Focus groups enable participants to discuss the topic with each other and to consider areas that they perceive to be important (Kitzinger, 1995).

### Participants

Purposeful sampling was used to recruit 23 parents/carers of children aged between 5 and 11 years who had ASD, DCD, and/or ADHD. The social media site TikTok was used for recruitment to gain a diverse range of participants from a broad geographic reach within the United Kingdom (UK). Table 1 outlines demographic information for the participants. In line with principles of participatory research, participants received a £30 retail voucher for their participation (Ham et al., 2004). Following an initial post on social media, the focus groups were filled within a day on a first come, first served basis, and a waiting list was then established. Participants were sent the consent form and participation information sheets, and then asked to complete a demographic questionnaire.

**Table 1.** Participant demographic information.

Characteristics	Details
Age range of parents	23–37 years
Highest level of parental education	Postgraduate degree (4), first degree (13), higher education diploma (3), HND/HNC (3)
Gender of parents	Women (16), men (7)
Ethnicity	Mixed (9), White British (9), Black (3), White other (2)
Diagnosis of a child	ASD (14), DCD (5), ADHD (3), ASD and ADHD (1)
Educational setting of child	Special school (10), home (9), mainstream school (4)
Gender of child	Boys (12), girls (11)
Age of child	5–11 years

HND: higher national diploma; HNC: higher national certificate; ASD: autism spectrum disorder; DCD: developmental coordination disorder; ADHD: attention-deficit/hyperactivity disorder.

### Focus groups

Data were collected through three online 90-minute focus groups between March and May 2023. Online groups were chosen as these can reduce barriers to participation such as time, cost and childcare requirements (Meadan and Dacewitz, 2015). In line with recommendations for focus group numbers, initially eight participants were recruited per focus group (Stalmeijer et al., 2014), and two focus groups were planned. However, in the first focus group, only four of the eight participants who were recruited joined online. Therefore, for the subsequent groups, additional participants were recruited from the waiting list. Only seven participants joined the second focus group (out of 11 recruited), so a third focus group was run in which all 12 of those recruited joined.

All focus groups were led by the same facilitator (TJ), a clinical academic occupational therapist. In each focus group, the facilitator led a brief ice-breaker activity where everyone was asked to say a personally meaningful occupation, to get participants comfortable with speaking, and to make sure that they understood what occupations were. The presenter then briefly presented a draft outline and rationale for an Occupational Therapy Parental Intervention programme based on the PEOP model (Baum et al., 2015). Supplemental 1 displays the presentation slides, and Supplemental 2 provides a transcript of the presentation. Then pre-developed semi-structured topic guides were used to gain parents’ views on the acceptability and feasibility of the intervention (Supplemental 3). All focus groups included a 10-minute break, and they were audio- and video-recorded and later transcribed verbatim for analysis.

### Data analysis

Data were analysed using Braun and Clarke’s (2006) six stages of thematic analysis: (1) TJ familiarised themselves with the data through re-reading the transcripts and making notes and pictures. (2) The data were then coded by TJ using an iterative process by means of Microsoft Word comments. Codes were closely linked to participants’ explicit accounts. Once all three focus groups had been coded, TJ transferred all codes alongside data extracts to an Excel spreadsheet. There were 50 codes in total, and these, alongside data extracts, were then reviewed by AB and discussed and refined together. (3) TJ then sorted codes into potential themes and subthemes, producing a set of candidate themes. (4) Candidate themes were reviewed with reflexive discussion by TJ and AB to consider if they captured the codes and data clearly and if they needed to be defined, collapsed or expanded. (5) AB and TJ then completed the analysis by defining and naming the themes and writing a narrative for each. In line with Braun and Clarke’s recommendation of theme and subtheme numbers to enable breadth and depth to the analysis, the final thematic map consisted of four themes. (6) The analysis was then written up in full.

Braun and Clarke’s (2006) thematic analysis is based on relativism, which asserts that knowledge is subjective. Researchers are unable to analyse data without drawing from their own positions and perspectives; therefore, researchers should aim to make their positions clear within their research (Braun and Clarke, 2021). TJ is an Occupational Therapist for Children and Young People as well as an Occupational Therapy academic. She also has Dyslexia, which is a neurodevelopmental disorder. AB is a Professor of Psychology whose research has focused on DCD, and she is also a parent and a grandparent and has worked extensively with children and their families. The range of research, clinical and personal experience aided reflexive discussions considering multiple perspectives. Throughout the process, TJ and AB engaged in reflexive discussions, and TJ kept a reflexive journal. Trustworthiness is enhanced through audio and video recording of the focus groups, the detailed spreadsheet aligning codes to data extracts, as well as a rich description of the data collection and analysis methods used.

Ethical approval for this study was provided by the Oxford Brookes University Cross-Faculty Research Ethics Sub-Committee, number: 231667.

## Results

Four main themes were generated from thematic analysis of the focus group data: (1) Parents want to develop skills to support their child, (2) parents also need support, (3) there are barriers to reaching parents and (4) parents need an inclusive environment to promote their learning. These are outlined below. Quotes from Focus Groups (FG) are labelled FG 1–3 according to which of the three focus groups the quote came from.

### Theme 1: Parents want to develop skills to support their child

Parents considered the overall idea of the intervention presented as useful. *‘It’s exciting as a parent to have something new come along that we can learn. . .and help make more difference’ (FG1).* Some parents also highlighted that they felt the intervention would ‘*help parents bond more with their kids*’ (FG3).

Parents generally seemed to understand the idea of the Person-Environment-Occupation Model and its application to parent education ‘*I think this theory has a lot to be able to assist, not just a child, but as a parent as well’ (FG2); ‘it’s an opportunity for parents to learn how to best take care of their kids’ (FG3).*

Parents highlighted some priority areas that they would like to develop skills in, including developing skills in their own self-regulation: ‘*what I would like to learn is, apart from the let’s just say the environment and the task, it’s probably a bit of patience, a bit of let’s just say self-awareness*’ (FG1); ‘*how do I calm myself?’* (FG1); ‘*how to speak to my child when I am angry*’ (FG1) ‘*so that I would not end up bullying the child*’ (FG1). Parents highlighted that they would like skills to ‘*help their child have more confidence’* (FG2) and *‘build their self-esteem and sense of accomplishment’* (FG3). They also wanted to develop confidence in themselves as parents ‘*it’s about telling parents that things are possible’* (FG2), ‘*you are not told how you can work with these barriers*’ (FG2). Parents also highlighted developing skills in how to support sibling relationships as a priority – *‘help him be more social with his siblings*’ (FG3) ‘*there might be some activities that might also involve being close to your siblings*’ (FG3).

### Theme 2: Parents also need support

Parents spoke about how challenging their role can be ‘*as parents it could be really difficult, it could be draining*’ (FG1), ‘*It can be very frustrating and discomforting*’ (FG2) ‘*I just want to cry*’ (FG1), ‘*I just want to shout’* (FG1). They highlighted their high stress levels ‘*right now I am very stressed*’ (FG2), ‘*we are all going through all the stress*’ (FG3); and that stress can ‘*take a toll of your health and mental wellbeing’* (FG3). Parents highlighted how alone they feel, ‘*no one is there for you*’ (FG2) ‘*no one even asks if we are ok*’ (FG3). Parents also spoke about the guilt that they feel, *‘I have got this all wrong, I am such a bad parent*’ (FG2); ‘*you have to live with the guilt*’ (FG3). Parents highlighted a lack of self-confidence in their ability; they felt that they ‘*do not know much*’ (FG1), ‘*we don’t actually know what to do’* (FG3) because there is ‘*no handbook*’ (FG2).

Parents emphasised that, as well as support for their children, they also needed support. Currently, they felt that ‘*most parents are not personally involved*’ (FG1) in OT, *‘you don’t know what’s going on. You don’t even know whether they’re progressing*’ (FG3). They highlighted that a positive of the proposed intervention is that it ‘*gets parents involved*’ (FG3). They felt that this intervention could empower them ‘ *I feel like it has saved me the stress, I know what to do, it is like a first-time manual that has never been made before’* (FG2). ‘*It’s going to impact the child positively as well as helping the parents to escape stress*’ (FG2).

Parents identified key features that they felt would be important in the group to further support their own well-being. Parents identified that ‘*oftentimes [they] did not get to meet up with other parents*’ (FG3), and they felt that this would be important. ‘*It should be more of a support network*’ (FG2), ‘*it is always better if people could share their experience*’ (FG3). They also highlighted that social contact could be facilitated ‘*every parent who decides to take part in this programme are allowed to have, like personal contact with other parents*’ (FG2). They suggested ways that socialising could be facilitated by ‘*having fun*’ (FG3), and *‘food [being] included in the programme*’ (FG2), as well as *‘dancing*’ (FG2), and ‘*15 minute break(s)’* (FG2). They also highlighted that the intervention could be beneficial beyond parents ‘*it needs to be on a broader scale’* (FG2), ‘*everyone should be interested*’ (FG1), ‘*friends*’ (FG1), ‘*caregivers and family members*’ (FG1), ‘*tutors*’ (FG2), ‘*neighbours*’ (FG3). Broadening the intervention could reduce the burden on parents, as many people would know how to best work with their child.

### Theme 3: There are barriers to reaching parents

Whilst parents acknowledged that the intervention could be beneficial, they highlighted barriers to reaching parents. They discussed that ‘*time is an issue*’ (FG3), and parents also identified that managing multiple responsibilities could cause a barrier. ‘*We are working parents’* (FG3), ‘*I have to fix dinner and lunch*’ (FG3), ‘*I have other kids as well*’ (FG2), ‘*mines all got health issues as well*’ (FG2). Parents also discussed the difficulty of splitting their time among their children ‘*it’s not like this is our only child*’ (FG3), ‘*as a parent all children need equal attention*’ (FG2), ‘*spending so much attention on them and just like how to take care of one kid would be something that a lot of people might not be interested in*’ (FG1). However, parents also commented that ‘*most parents also try their best to be available*’ (FG3).

Cost was also raised as a potential barrier, both in terms of ‘*the cost of the programme*’ (FG2) and associated costs such as ‘*transport costs*’ (FG3). There was also anxiety expressed around managing the workload and understanding the content, ‘*is it something I could cope with?*’ (FG1). Stigma was also identified as a barrier: ‘*through diagnosis that there [is] such a stigma*’ (FG2) as well as ‘*stigma from other people in the general life*’ (FG2). A parent said that this stigma might prevent a parent from accessing a group, ‘*they don’t want to bring their kids to it in case it’s seen as if they are doing something wrong*’ (FG2). Parents said that when deciding to join, they ‘*think of the risks, the barriers, the merits, wherever you gain, and is it worth it?*’ (FG2).

Parents emphasised that the programme needed to be ‘*very flexible*’ (FG2). Some parents suggested that if ‘*it’s on a weekend that might be helpful*’ (FG3). Parents also discussed how online sessions could be more accessible compared to face-to-face sessions ‘*virtual will actually help to reduce the cost*’ (FG3), ‘*online is quite flexible*’ (FG2), ‘*it gives parents the time to do other things while they’re in the meeting*’ (FG2). However, parents also noted the importance of in-person sessions ‘*I feel I could connect more with people physically*’ (FG3). They suggested ‘*a mix of both*’ (FG2) could work well, either ‘*one physical session, then one virtual session*’ (FG2) or ‘*options for both*’ (FG3) that parents can choose from.

### Theme 4: Parents need an inclusive environment to promote their learning

Parents discussed the importance of an inclusive environment. They highlighted that there are parents who ‘*may be disabled*’ (FG3), including ‘*parents themselves, [who] are autistic*’ (FG3), as well as those who could be ‘*physically challenged*’ (FG3) or who ‘*might not be able to understand perfectly*’ (FG3). Parents also highlighted ‘*racism and microaggression in the system*’ (FG3) as a barrier that would need addressing, ‘*it’s easier for a white parent to look down on a black parent*’ (FG2) ‘*same thing goes with the professionals*’ (FG2).

Parents proposed several ways in which the proposed intervention could embed inclusive approaches. They highlighted the need for racial diversity in participants, the professionals and organisers of the intervention ‘*mixture of different races as the teachers*’ (FG3), ‘*a mixture in the organisers*’ (FG3), ‘*people taking back the feedback from people should not just be of one race, I think [they] should be of different races*’ (FG3). They also suggested ‘*incorporating a bit of our culture and beliefs*’ (FG3) into sessions, ‘*it can be your food, your dress code or tell a bit of your history*’ (FG3). Some parents suggested that being online could reduce the effects of racism ‘*no one will come and say, okay, he shouldn’t sit here*’ (FG2). Parents also highlighted the need for explicit ‘*ground rules*’ (FG2) ‘*a guideline or principles, the do and the don’t*’ (FG2), suggesting that these will support people to work well alongside each other.

Parents emphasised the importance of ‘*accessibility*’ (FG2) both in terms of physical space ‘*it needs to be a very spacious area*’ (FG3) and the need to potentially adapt information into ‘*images and proper graphics*’ (FG3) to support people to learn or have alternative communication options ‘*If I can’t communicate in English, is there the option to choose?*’ (FG3). Parents suggested that getting parents ‘*demographics before*’ (FG3) the group can help professionals to plan to ensure inclusivity in the sessions. Parents also stated that it would be ‘*really important that we get people who have patience*’ (FG2), to run the group, ‘*get someone who can talk to [parents]*’(FG1). They also highlighted that ‘*some sort of support system in the background*’ (FG2) from professionals could support participation and understanding. It was highlighted that those ‘*who have been taught might really lack that skill at some point*’ (FG3) and ‘*a line of contact which one can call*’ (FG3) could support the learning for all parents.

## Discussion

This study sought to gain the perspectives of parents/carers of children with neurodevelopmental disorders on the acceptability and design of a novel parental educational intervention underpinned by OT theory. It was found that parents felt that this new intervention could be useful for both their child and themselves. Using thematic analysis, four themes were identified: (1) Parents want to develop skills to support their child, (2) parents also need support, (3) there are barriers to reaching parents and (4) parents need an inclusive environment to promote their learning.

### Parents want to develop skills to support their child

Parents highlighted a desire to learn skills to better support their child. Specifically, parents mentioned a desire to improve their own emotional regulation skills. Parents felt that lacking these skills could sometimes lead them to respond more harshly to their child. This aligns with Lunkenheimer et al. (2023) who argue that there is a link between poor parental emotional regulation and harsher discipline, which could negatively impact children. Whilst teaching emotional regulation skills to children who have neurodevelopmental disorders is currently a common form of therapy (Cai and Uljarević, 2021), it is not common practice to support parents to develop skills in the same area despite the impact this could have on both the parent and child. Parents also reported a desire to develop skills to support their child to have better sibling relationships. Research on the participation of parents and children with DCD in telehealth OT also found that parents valued the opportunity to involve siblings in their child’s therapy (Bourke et al., 2023). This suggests that parents would really value occupational therapists addressing sibling relationships as part of their practice.

### Parents also need support

Parents reported that they were experiencing high stress levels. This aligns with research which has found that parents of children with neurodevelopmental disorders have higher stress levels compared to parents of typically developing children (Craig et al., 2016). Long-term stress can lead, both directly and indirectly, to poorer overall physical and psychological health outcomes (O’Connor et al., 2021). Therefore, it is important that the high stress levels of parents of children with neurodevelopmental disorders are addressed. Parents felt that having an intervention that enabled them to connect with other parents could support them to feel less stressed and better connected. Social support has been found to have a moderating effect on parental stress and depression (Park and Lee, 2022). Parents highlighted creative suggestions such as the use of food and dancing to bring people together during the intervention, as well as the importance of encouraging the sharing of contact information.

Parents also mentioned that a lack of confidence in how to support their child increased their feelings of stress. They noted that when their children were having therapy that they often felt excluded and were unsure what was being done in sessions. These experiences are in direct contrast to current best practice in children’s OT, which is to work in a family-centred way, including parents in the OT process (American Occupational Therapy Association, 2020). This highlights the gap between the creation of evidence-based recommendations and their adoption into everyday practice, which is reported to be 17 years (Morris et al., 2011).

### There are barriers to reaching parents

Parents highlighted potential barriers to attending the course, which included time, the cost of both the course and the travel to attend, stigma, as well as juggling multiple responsibilities such as work and other children. Parents discussed the difficulties and mixed feelings that accompany spending dedicated time to support only one of their children. Discomfort with singling out time with one child over their siblings has also been highlighted by parents of children with DCD engaging in telehealth OT (Bourke et al., 2023). Parents felt that flexibility within the new parental intervention could help support them better manage competing responsibilities. This included the potential for virtual sessions and sessions run on the weekend. As the focus groups for this research were run online, all participants had access to technology, which would enable them to access virtual sessions; however, 5% of households in the UK do not have access to the internet (Ofcom, 2025). Therefore, it is important to consider that virtual sessions would not be accessible for all.

### Parents need an inclusive environment to promote their learning

Parents highlighted the need for the parental intervention group to be inclusive. Disability was highlighted as an area that needed to be considered carefully to ensure that parents were not excluded from participating. Parents highlighted the need to gain key demographic information from participants to adequately plan adjustments, as well as consider aspects of universal design (e.g. having a space large enough for those with physical disabilities). In particular, parents highlighted the increased likelihood that parents of children with neurodevelopmental disorders are more likely to experience a neurodevelopmental disorder themselves (Gidziela et al., 2023). Occupational therapists are well placed to consider how to design inclusive spaces and should be considering this at all times, and not only when designing groups specifically for those with disabilities.

Parents also highlighted the need for cultural and racial inclusivity, discussing experiences of racism from group members and professionals in the past. The Health and Care Professions Council (HCPC, 2023) has recently updated the standards for occupational therapists’, including a greater emphasis on the need to ‘recognise the impact of culture, equality and diversity on practice and practice in a non-discriminatory and inclusive manner’. This aims to address occupational therapists often not seeing or attending to the experiences and inequities faced by black and marginalised populations (Ahmed-Landeryou, 2023). Participants also highlighted the need for the organisers of the group to be racially diverse, as well as having clear ground rules within the group and opportunities to share aspects of their culture with each other.

### PEOP model

The parental education intervention aims to guide parents in how to use the PEOP model to support their children’s occupational participation (Baum et al., 2015). However, parents also highlighted the importance of using the PEOP model more broadly within the creation and design of the intervention. Parents highlighted the heterogeneity of the parental experience and raised important considerations aligned with the PEOP model for the design of the intervention itself. They discussed the need to consider the skills of the parent/carer, their environment, their own occupational demands, and the impact that these can have on participation.

## Strengths and limitations

A strength of this research is the racial diversity within the sample, which has likely impacted the topics raised by participants. Often, health research has included predominantly participants of white ethnicity, and the importance of including those from minoritised backgrounds has been raised as a priority (Routen et al., 2022).

Parents did seem to generally understand the idea of the PEOP model, and its application to parent education, and considered the overall idea of the intervention presented as useful. However, beyond acknowledging the benefits of the idea to help parents and their children, most of the discussion focused on general considerations for parental education intervention rather than considering the unique contribution of the intervention presented. This could be because of the limited time available to explain the application of the PEOP theory, or it could also be explained by many of the parents reporting that they did not have experience of OT intervention. This would limit their ability to evaluate the uniqueness of this intervention method compared to others.

A potential limitation of this research was the use of virtual rather than face-to-face focus groups. Despite multiple requests for participants to have their cameras on during the focus group, only a minority did, which potentially impacted the fluidity of the interactions with and between participants. It was also more challenging to verbally engage quieter participants, who often instead chose to write their response in the chat. However, participants did disclose sensitive and personal information, and this could have been supported by the anonymity provided by the online setting in which their camera was off.

## Conclusions

Aligned to RCOT (2019) research priorities, parents/carers highlighted that a new parental education intervention could enable occupational therapists to work more effectively with families. They saw the benefits of learning how to use the PEOP model (Baum et al., 2015) to support them in independently problem-solving everyday challenges to occupational performance for their children. They also highlighted potential challenges to their participation in the intervention and expressed the importance of considering the needs of parents/carers when creating it. This emphasises the importance of not only underpinning the educational content with the principles of the PEOP model but also using the PEOP model within the design of the intervention to consider the needs of parents/carers themselves.

**Key findings**

- Parents seek practical skills to support children and regulate their own emotions.
- Parents require emotional support, peer connection and inclusive learning environments.
- Barriers include time, cost, stigma and accessibility constraints.

**What the study has added?**

This study advances OT practice by evidencing the value of co-produced, theory-driven parental education interventions, highlighting inclusivity, empowerment and family-centred approaches as essential for service innovation.

## Supplemental Material

sj-docx-1-bjo-10.1177_03080226251404423 – Supplemental material for The perspectives of parents/carers on a new parental education occupational therapy intervention Supplemental material, sj-docx-1-bjo-10.1177_03080226251404423 for The perspectives of parents/carers on a new parental education occupational therapy intervention by Teresa Joyce and Anna L. Barnett in British Journal of Occupational Therapy

sj-docx-2-bjo-10.1177_03080226251404423 – Supplemental material for The perspectives of parents/carers on a new parental education occupational therapy intervention Supplemental material, sj-docx-2-bjo-10.1177_03080226251404423 for The perspectives of parents/carers on a new parental education occupational therapy intervention by Teresa Joyce and Anna L. Barnett in British Journal of Occupational Therapy

sj-pdf-3-bjo-10.1177_03080226251404423 – Supplemental material for The perspectives of parents/carers on a new parental education occupational therapy intervention Supplemental material, sj-pdf-3-bjo-10.1177_03080226251404423 for The perspectives of parents/carers on a new parental education occupational therapy intervention by Teresa Joyce and Anna L. Barnett in British Journal of Occupational Therapy
