# Supplementary material for: The perspectives of parents/carers on a new parental education occupational therapy intervention
Source: Br J Occup Ther. 2025 Dec 26;89(7):468–75. doi: 10.1177/03080226251404423 (PMC13310313; doi:10.1177/03080226251404423)
Supplement: sj-docx-1-bjo-10.1177_03080226251404423 – Supplemental material for The perspectives of parents/carers on a new parental education occupational therapy intervention [file sj-docx-1-bjo-10.1177_03080226251404423.docx]

**Supplementary Information 2 Transcript of the presentation to participants**

This is a transcript of the presentation to the participants of the third focus group on 15/05/2023. All focus groups received the same presentation

Presentation Transcript

What I’m going to do now is share a presentation with you, that shows you the idea of this overall intervention that we have. I'm going to ask you some questions during it, but I’m going to ask you most of the questions after it. But again, as I said before, if people could have their cameras on, that would be really helpful, because it's really challenging, talking to empty screens, because I don't know if you can hear me, or if you understand what I’m saying, and it'll feel similar with each other when you are having a discussion, because we're trying to create a conversation. So what I'm going to do is I’m going to share my screen.

This is the idea for a new parental education intervention. But first, it's very important that we think about what is occupational therapy, because I know that some of you might have had experience with occupational therapists before, and some of you might not have. So Occupational Therapists work with people to enable them to participate in activities that they need or want to do. And we split these activities into 3 main areas. So the first area is self care, and this is anything that we do to look after ourselves, like, for example, having a bath or a shower, washing our hair and making a meal for ourselves. And then we have productivity. So for children, this is often going to school, or going to nursery, or going to university as they get a bit older. So, things related to education and the tasks and activities that are necessary at those times. Things like concentrating in class handwriting, packing your bag for school, those types of things. And as people get older, Occupational Therapists are interested in employment, and all of the tasks that are involved in employment. And then we're also interested in leisure, which is why I was asking you at the beginning about the types of things that you like to do for fun. So, as people said, things like dancing and singing and playing sports. And so we are interested in supporting people to be able to engage in these leisure activities as well.

Question to participants and some discussion

Presenter: *So the first question I have for you is, what is your experience of Occupational Therapy for your child? So far have you had experience of Occupational Therapy, or is this something that is new to you?* I’ll get the chat up in case people are in the chat, so feel free to write in the chat, or to speak out loud. Have you had Occupational Therapy for your child before?

[Presenter reads some responses from the chat]

Presenter: So P23 says yes, P23, what did that look like? What types of things were involved?

It's new to P19.

P23 said. “It was okay, for her self-care”. Was it the Occupational Therapist working directly with your child? Or did you go to a group where they taught you certain things as a parent?

It's new to P17.

So, P23 had occupational therapy working directly with her child. Fantastic. Thank you, P23.

And P21 What about you? Have you had occupational therapy for your child.

P15: I have

Presenter: P15, you have. What did that look like?

P15: The therapist worked directly with my child and I was fully involved as most of the tasks we had been given I had to see through them at home to make sure that he was able to carry out the tasks on his own and most of the times when we had dr appointment she would try to see how far we had gotten as a team. So she would try to go over the stuff with him, and it was so much easier that way.

Presenter: Fantastic. Thank you so much. P15 and P20 and P22 have also said that it's new to them. So we've got a bit of a mix in the room of experience. P13 What experience have you got of Occupational Therapy? If you're talking, we can't hear you. So P21 has said that it's new to him but that it was recommended.

Presenter: Okay, if anybody that hasn't contributed so far. If you could write in the chat when I'm going through this presentation, just to say, if you have had Occupational Therapy in the past, that would be really helpful, because it'll give us an idea of the types of experience people have with Occupational Therapy so far. So how do Occupational Therapists enable participation like I was just saying that we do?

Presentation Continued

Presenter: So, this that you can see right now is a picture of the person, environment, occupation, and participation model. Occupational Therapists use this model to support their reasoning. Occupational Therapists consider the skills of the person, the demands of the activity that they're trying to do, and also the environment in which they're trying to do the activity And if there is a good match between the skills of the person and the task they're trying to do, and the environment in which they're doing it in then participation is likely to occur.

But if there is not a good match between the skills of the person, the tasks they're trying to do, and the environment in which they're trying to do it in, then there is not a good match between these things, and participation is less likely to occur. Like, for example, somebody said at the beginning that they like to play football. I can't remember exactly who I’m so sorry, but I’m imagining that you are a really good footballer and that when you're in the park with your friends, you are a striker and your scoring loads of goals, and it's fantastic. And that's because your skills as a footballer are really well matched to the activity, which is playing football with your friends, and the environment, which is your local park that has a football pitch in it. But if I was to suddenly pick you up and put you into the Premier League, and you had to play football in the Premier League, then it's unlikely that you will participate in the same way that you do in the park, and you might not even touch the ball, because your skills now no longer match up with the activity that we're asking you to do, which is now Premier League football, and the environment in which you'll be doing it in, which is a stadium full of people staring at you and potentially live television cameras. So suddenly participation no longer is likely to occur.

Here's another example - if you had a child who had basic bike riding skills, and they wanted to do the activity of riding their bike and they wanted to ride their bike up and down a path in the environment of a quiet park, there's a good overlap between the skills of the child, which is basic bike riding skills, the task they're trying to do, which is riding their bike up and down, and the environment in which they're trying to do it, in, which is a quiet park without many obstacles or business. So, therefore, participation is likely to occur there. However, if you take that same child who has basic bike riding skills, and instead of them just cycling up and down a path, they had to go on a path that was more curved, and it was steep, and up and down the hill, and the environment that they were in was actually very busy, full of pedestrians and full of other cyclists. Then participation is much less likely to occur, because there's no longer a good overlap between their basic bike riding skills and the bike riding task that they set themselves, and the environment in which they're trying to do this bike riding task; so it's less likely to occur. Does that generally make sense to people?

P15: Yeah, it does

P17: Yes, it does

Presenter: Excellent. Thank you. I'm really glad, because it can be quite a challenging thing to try to communicate. But if somebody doesn't understand, please do let me know, and I can try to explain it in a different way.

Okay. So in Occupational Therapy what we do is we problem solve everyday challenges by thinking about the best ways to build up the skills of the child or the person that we're working with, but in this instance, we're talking about children. So how to best build up the skills of the child so that they're able to do the things that they need and want to be able to do. We also think about how we can adapt aspects of the task or activity that they're trying to do, and also how we can change elements of the environment so that they're able to participate at their current level of skill as well. And this is the reasoning process that we use, and it's also a reasoning process that could potentially be taught to parents.

There is research evidence that has found that parental education can be effective in supporting children's participation in everyday activities, as well as supporting parental well-being. But the parental education interventions that exist at the moment are quite narrow in their focus. So, they're looking at particular areas, like, for example, communication skills or behaviour management, or they're teaching parents mindfulness skills in isolation. There is not currently a parental education intervention that's underpinned by the Occupational Therapy theory that I just explained to you and I think that teaching parents how to think about using a person, environment, occupation, participation model, which is the model I just showed you could enable parents to be able to problem-solve the challenges to everyday life and this would enable parents to support their child's participation in lots of areas of everyday life rather than just going to an education group that's telling them how to tackle one specific area at the time.

So I’d like to say thank you so much for listening to me so far, and I’d really like to hear your thoughts about this intervention. So I’m going to stop sharing my screen in a second, and I would please like to know your thoughts.
