# Supplementary material for: The perspectives of parents/carers on a new parental education occupational therapy intervention
Source: Br J Occup Ther. 2025 Dec 26;89(7):468–75. doi: 10.1177/03080226251404423 (PMC13310313; doi:10.1177/03080226251404423)
Supplement: sj-docx-2-bjo-10.1177_03080226251404423 – Supplemental material for The perspectives of parents/carers on a new parental education occupational therapy intervention [file sj-docx-2-bjo-10.1177_03080226251404423.docx]

**Supplementary Information 3: Draft Questions for the focus group**

**Part 1 – Warm up get to know each other**

Let’s get to know each other, please introduce yourself by saying your name (or a pseudonym if you would prefer), where you are from and something that you like to do for fun? I’ll go first.

**PART 2– Focused on the need and acceptability of the intervention**

1. I’d really like to hear your thoughts about this intervention idea

2. Tell me how you think this compares with what is currently available in OT intervention. To what extent do you think something like this might be needed?

3. What benefits/positive features do you think there might be with this intervention? (picking up from responses to (1)).

4. What issues or barriers do you think there might be with this intervention? (picking up from responses to (1)).

5. How would you respond if something like this was available/on offer?

**Part 3 – Focused on electing ideas around key aspects of the programme**

1. What frequency and duration of individual sessions do you think is feasible for parents/careers and why?

2. What duration of the programme (in weeks) do you think is feasible for parents and why?

3. Do you think parents/carers would prefer Online or in Person sessions and why?

4. Tell me what you think would be important to consider/include in this programme. I’m interested to hear if you think there is anything important that should be included in this programme.
