# Supplementary material for: The perspectives of parents/carers on a new parental education occupational therapy intervention
Source: Br J Occup Ther. 2025 Dec 26;89(7):468–75. doi: 10.1177/03080226251404423 (PMC13310313; doi:10.1177/03080226251404423)
Supplement: sj-pdf-3-bjo-10.1177_03080226251404423 – Supplemental material for The perspectives of parents/carers on a new parental education occupational therapy intervention [file sj-pdf-3-bjo-10.1177_03080226251404423.pdf]

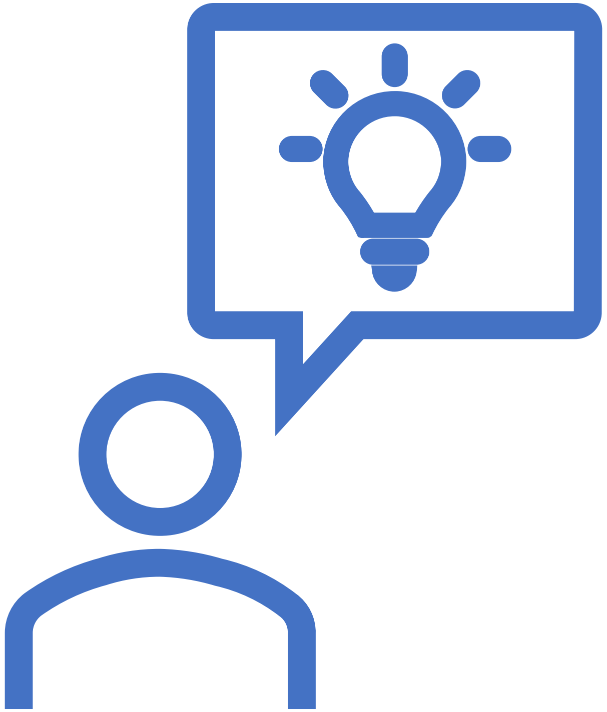

Idea for a New Parental Education Intervention

What is Occupational Therapy?

- Occupational Therapists work with people to enable them to participate in activities that they need or want to do

- We split these activities into the areas of:
  - Self-care – e.g. washing hair, making meals
  - Productivity - e.g. education, employment
  - Leisure – e.g. hobbies, socialising

How do Occupational Therapists enable participation?

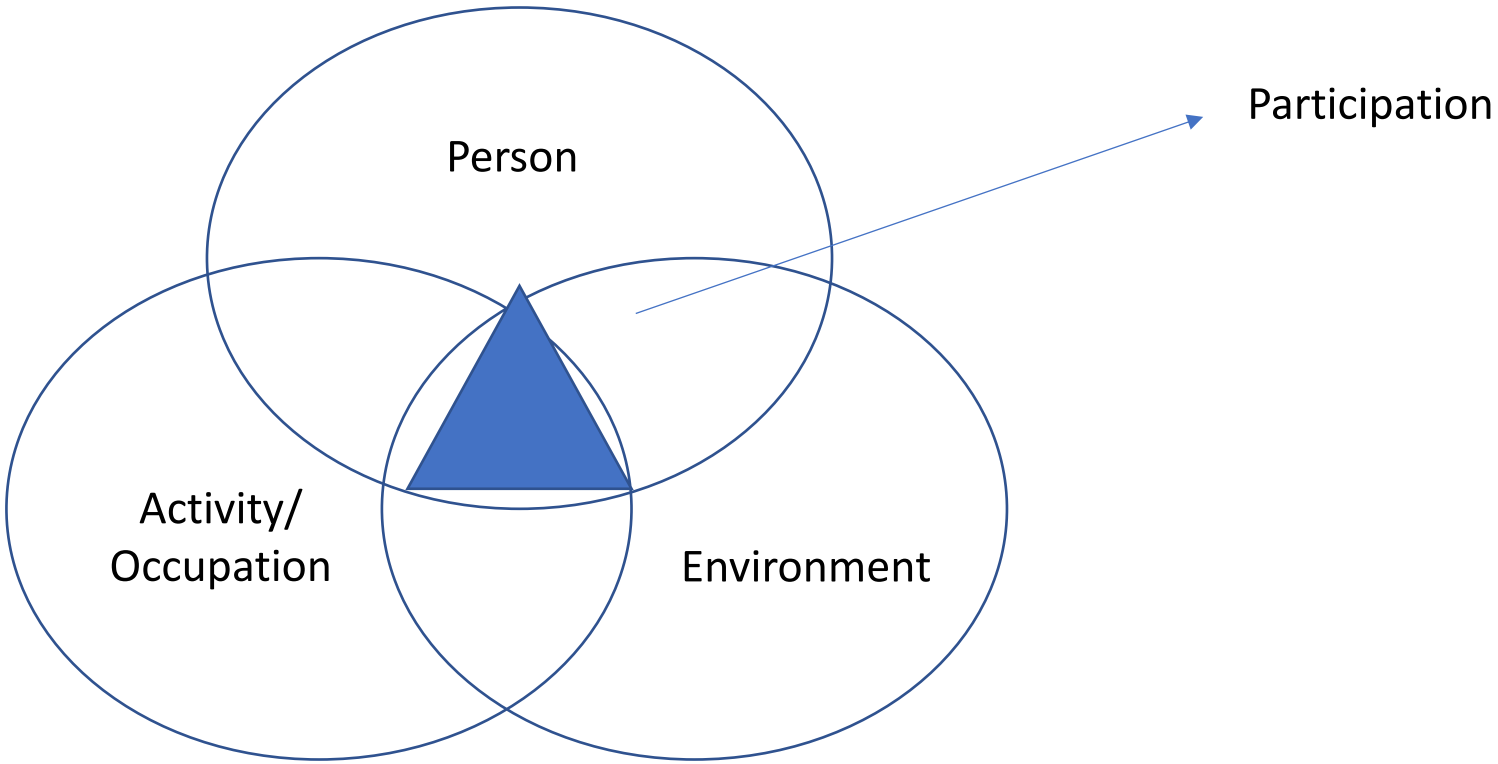

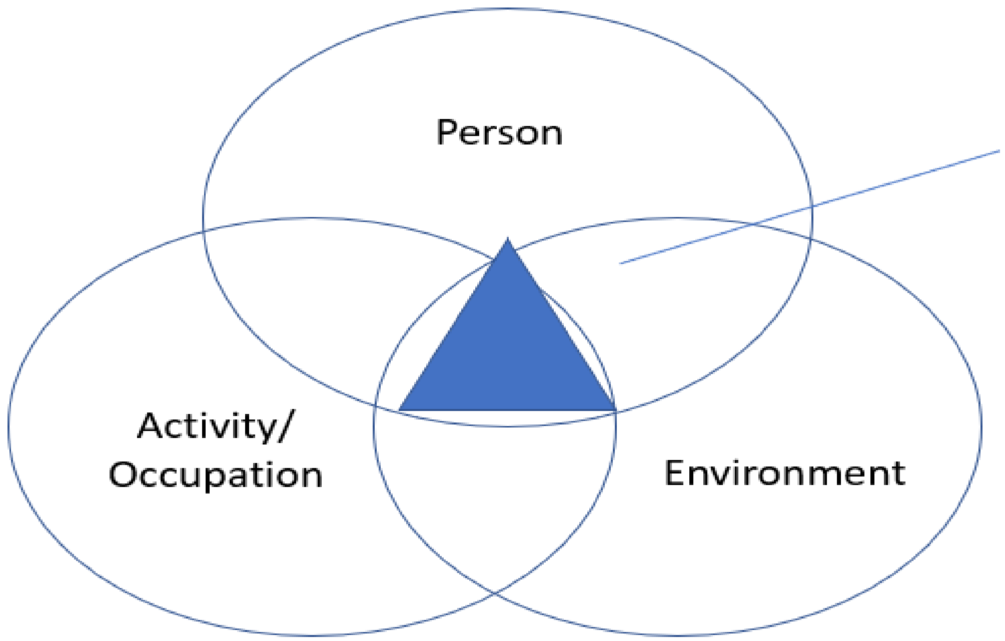
Person
Activity/
Occupation
Environment

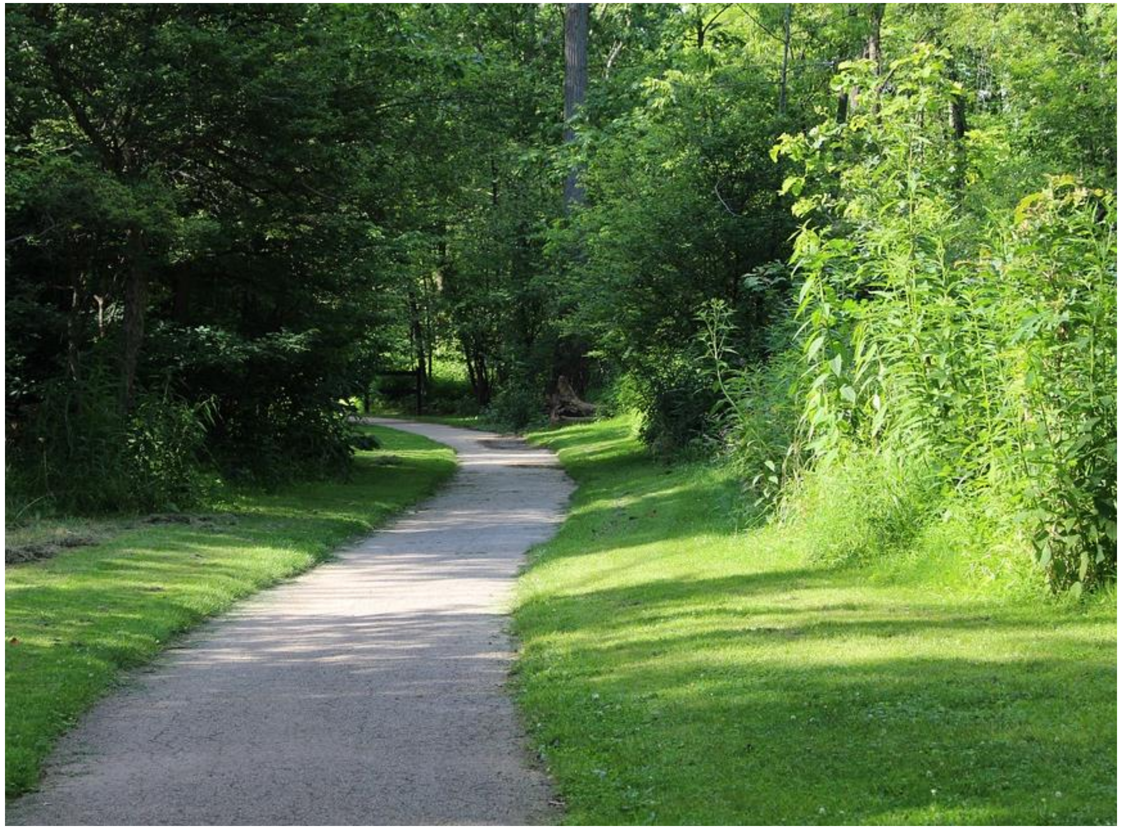

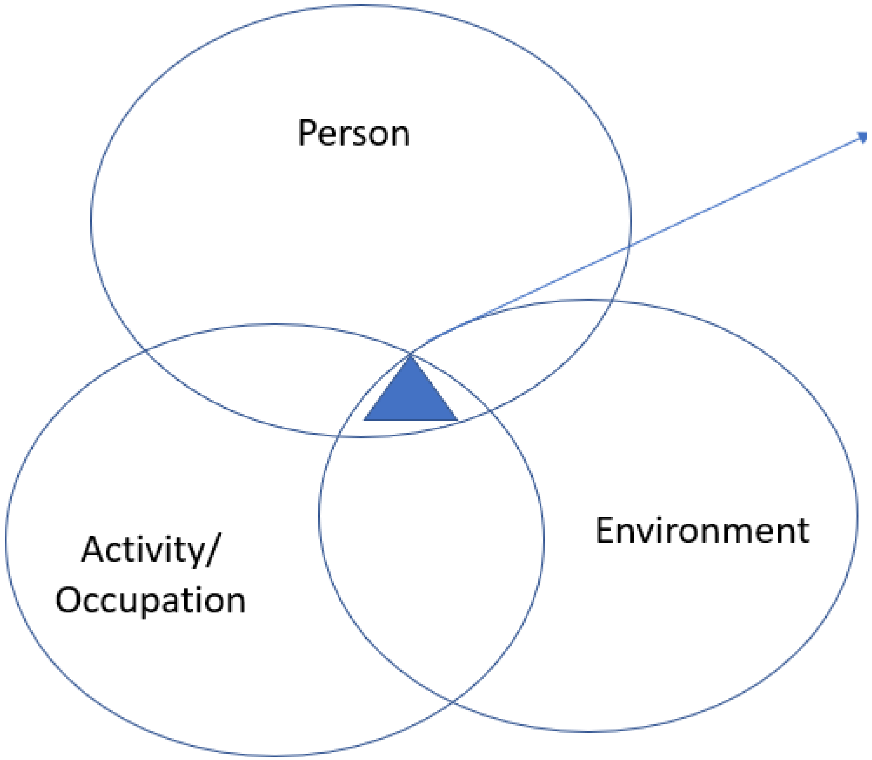
Person
Activity/
Occupation
Environment

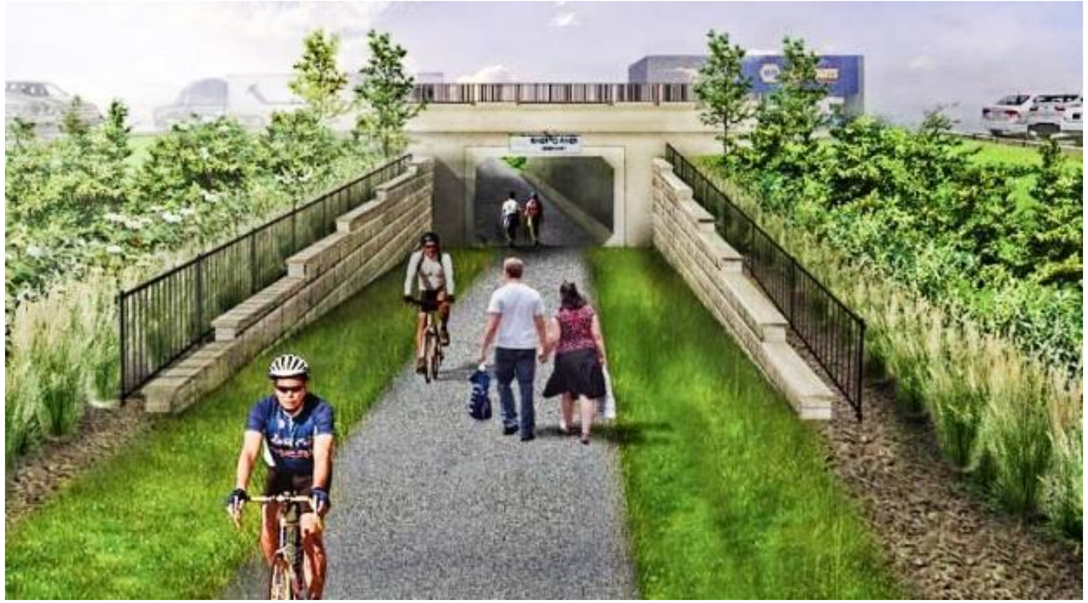

Occupational Therapy Intervention

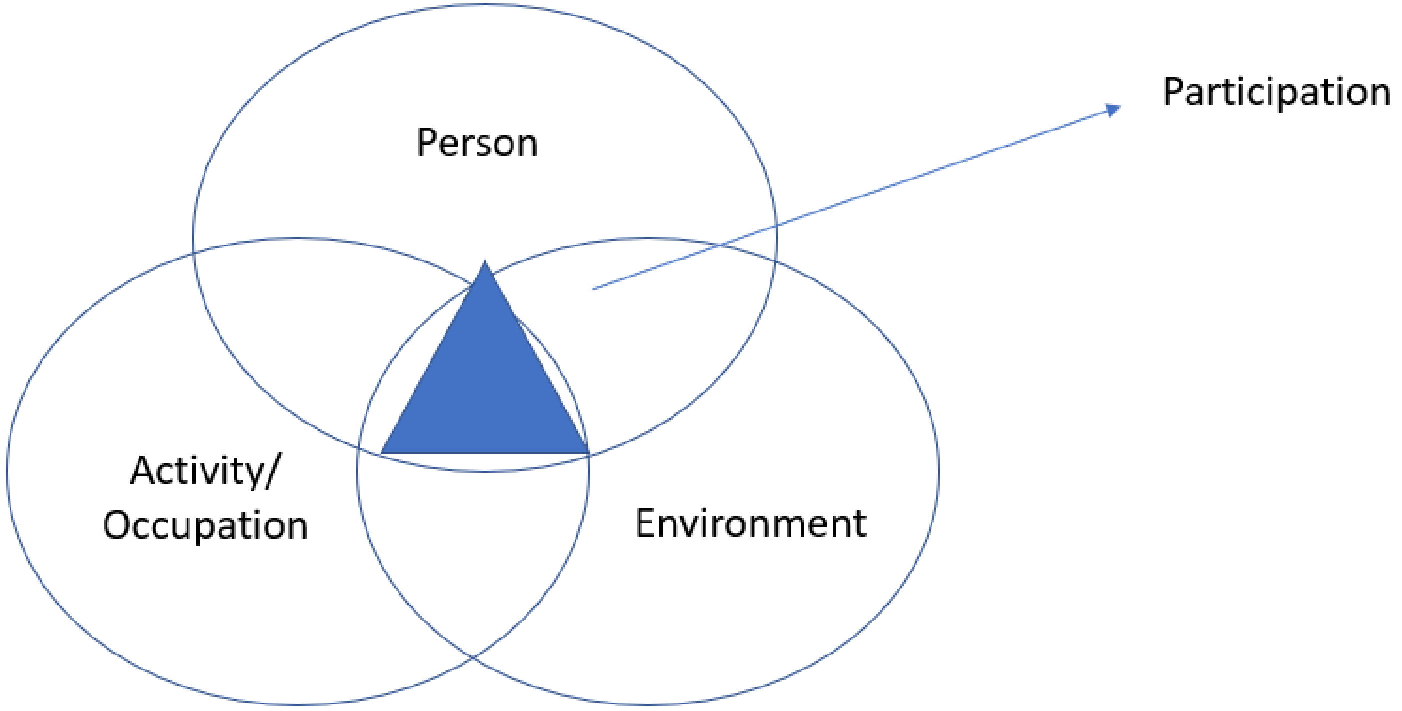

Parental Education Intervention

- Research evidence has found that parental education can be effective to support children's participation in everyday activities as well as supporting parental wellbeing.
- Current parental education interventions exist focused on:
  - Communication skills
  - Behaviour management
  - Parental Mindfulness

New Parental Education Intervention Idea

- There is currently no parental education intervention underpinned by Occupational Therapy Theory
- Teaching parents how to think about using the Person, Environment, Occupation, Participation model as well as how to follow the Occupational Therapy process could enable parents to assess and problem solve challenges to everyday life.
- This would enable parents to support their child's participation in all areas of their everyday life rather than one area at a time.

Thank you for listening

I’d really like to hear your thoughts about this intervention idea
